# ASGE Endoscopy Live: Management of Achalasia. A case of “anti-reflux POEM” in a patient with type II achalasia

**DOI:** 10.1016/j.igie.2023.11.006

**Published:** 2023-11-29

**Authors:** Stavros N. Stavropoulos, Aymen Bukannan

**Affiliations:** John D. Archbold Memorial Hospital Gastroenterology Group Thomasville, Georgia, USA

The American Society for Gastrointestinal Endoscopy (ASGE) has organized multiple live endoscopy courses with focus on different aspects of diagnostic and therapeutic endoscopy. The *ASGE Endoscopy Live: Management of Achalasia* course occurred on April 20, 2023, and included 3 live peroral endoscopic myotomy (POEM) procedures from 3 U.S. centers demonstrating different techniques. We demonstrated our “anti-reflux POEM” (AR-POEM) technique. The AR-POEM technique offers the possibility of individualized therapy. In our practice, for example, we offer conventional POEM to end-stage patients in whom maximizing esophageal emptying to avoid esophagectomy overrides GERD concerns; we reserve AR-POEM for patients with early disease and concerns about GERD/proton pump inhibitor use or a high predicted risk for post-POEM GERD (eg, high body mass index, hiatal hernia).

## Case presentation

A 62-year-old man presented with dysphagia for the past 2 years. He reported regurgitation and coughing episodes with sputum tinged to the color of his previous meal, occasional chest discomfort related to “feeling full,” and no weight loss. His Eckardt score was 6 (dysphagia, 2; regurgitation, 3; chest pain, 1; and weight loss, 0). Barium esophagram revealed delayed passage of contrast with narrowing at the esophagogastric junction (EGJ) and mild distention of the esophagus (maximal diameter, 4 cm), findings consistent with achalasia. High-resolution manometry revealed all swallows to be aperistaltic with 100% panesophageal pressurization and an integrated relaxation pressure of 26.6 mm Hg, findings consistent with type II achalasia. The patient had initial treatment with botulinum toxin injection, with limited response. He was referred for POEM. We elected to perform an AR-POEM technique to minimize post-POEM GERD as supported by our recently reported outcomes of 116 AR-POEMs propensity score matched to 116 conventional POEMs: at a minimum follow-up of 2 years (median of 34 and 55 months in the AR and conventional groups, respectively), 76% of patients completed pH/EGD objective GERD assessments.^1^ We found a significant reduction in GERD in the AR-POEM group (28% vs 67% positive pH studies [*P* < .001]; mean acid exposure time, 4.5% vs 10.3% [*P* < .001]).

## Brief description of the live endoscopy video

We performed a live AR-POEM procedure as part of an *ASGE Live Endoscopy* course that was focused on the treatment of achalasia. The procedure involved the following steps: (1) pre-POEM endoluminal functional lumen imaging probe measurements (EndoFLIP; Medtronic, Minneapolis, Minn, USA), esophageal landmark measurements, and planning of POEM orientation and length; (2) mucosal incision; (3) submucosal tunnel initiation and extension; (4) myotomy; (5) double-scope transillumination technique to assess the length and orientation of the gastric myotomy; (6) post-POEM EndoFLIP measurements; and (7) closure with endoscopic suturing (Supplementary Video 1, available online at www.igiejournal.org).

## Procedural and clinical outcomes

The AR-POEM procedure was performed successfully and transmitted in its entirety with a total transmission time of 45 minutes. There were no procedure-related adverse events. The patient was discharged home on postoperative day 1 after a barium esophagram showed no leaks and excellent esophageal emptying. The patient was stable with no pain and was discharged on a twice-daily proton pump inhibitor to be maintained until a planned pH study at 3 to 6 months post-POEM. The patient was also to remain on a modified diet of full liquids for 5 days and soft diet for 5 days. The patient’s Eckardt score at 1 month was 0.

## Technical highlights

### Important landmarks and spatial relations

The Z-line is usually located 1 cm distal to the start of the high-pressure zone. Also, the terminus of the tunnel and myotomy (assuming a 2-cm gastric myotomy) is predictably located 4 to 5 cm distal to the start of the high-pressure zone, which is easily identifiable without the need to insert the endoscope beyond it and risk injury of the mucosa that will overlie the POEM tunnel.

### Planning of the POEM strategy

A myotomy length of 6 to 7 cm is adequate for patients with type II achalasia (2- to 3-cm esophageal length, 2- to 3-cm high-pressure zone/sphincter, 2 cm gastric myotomy). A posterior approach may offer easier access to the submucosal space and easier closure, but there is significant vascularity, especially in the cardia. The orientation and site of entry are also affected by anatomic considerations and mucosal vascularity. For example, in this patient, a 6 o-clock orientation allowed us to plot a course that would allow identification and preservation of the oblique (sling) muscle fibers of the lower esophageal sphincter (LES), the hallmark of our AR-POEM technique, but also avoid a healing food impaction ulcer located at 7 to 8 o-clock and a small epiphrenic diverticulum located at 4 to 5 o-clock.

### Initial submucosal injection

It is important to avoid inadvertent deeper injection in the muscle or adventitia. This often appears as a relatively diffuse opaque bleb (rather than the sharp vertical translucent bleb shown in the video) and tends to occur when there is submucosal fibrosis. It may result in inadvertent tunnel initiation in the mediastinal space rather than the submucosal space (perforation).

#### Tips to avoid bleeding at the initial mucosal incision

There are several ways to avoid bleeding at the initial mucosal incision: (1) avoid mucosal vessels (subtle but identifiable) when injecting the initial bleb of saline; (2) use DRY CUT current rather than ENDO CUT (both, Erbe Elektromedizin GmbH, Tübingen, Germany); and (3) initiate the mucosal incision with an initial exploratory 1- to 2-mm puncture rather than a linear cut. This initial entry is blind to any superficial submucosal vessels. Accidental injury to a vessel can be easily coagulated within a small puncture site with spray coagulation. This is more difficult to accomplish within a linear cut in which the exact bleeding site can be harder to ascertain.

A final tip to avoid bleeding at the initial mucosal incision is to extend the puncture entry to a transverse incision. We perform transverse rather than longitudinal incision because we prefer closure with suturing rather than endoscopic clips. A slow lateral movement of the knife with DRY CUT current allows coagulation of superficial vessels

#### Important tips for the initial mucosal incision and entry into the submucosal space

The first tip for the initial mucosal incision and entry into the submucosal space is to dissect near the muscle rather than the mucosa (unlike endoscopic submucosal dissection) to preserve a thick submucosal layer on the underside of the tunnel mucosa. This structurally robust submucosa will bolster the overlying mucosa and mitigate against tearing of the tunnel opening during scope manipulation (particularly important in anterior POEM) and allow more secure closure. Second, a small 10-mm orifice is sufficient. Entry can be accomplished by using the “water pressure technique” as shown in Supplementary Video 1. In posterior POEM entry, another trick for entering the submucosal space consists of torquing the endoscope 180 degrees and using the up-angulation (rather than the weaker down-angulation) of the endoscope to enter the submucosal space (at which point the torque can be relaxed to the original orientation).

#### Tunnel dissection tips

We offer several tunnel dissection tips. First, the hybrid knife may achieve faster dissection with fewer bleeding episodes compared with knives without high-pressure injection capability. It is particularly useful in cases with severe fibrosis. We prefer the I-type hybrid knife, which allows more precise dissection than the T-type, in which the disk limits visibility of the dissection field directly in front of the knife. Second, we generally use DRY CUT current for dissection of avascular submucosa and preciseSECT current (VIO 3; Erbe Elektromedizin GmbH, Tübingen, Germany) for vascular submucosa (for small vessels). Third, to avoid tunnel meandering, we present on the video a technique that we had published consisting of placing a mark on the shaft of the endoscope that allows continuous monitoring of the tunnel orientation and corrective actions as needed.

#### Hemostasis tips

All the techniques discussed here are shown in Supplementary Video 1. For small arteries (≤1 mm) and pre-emptive coagulation, use broad contact of the knife with very low power Forced or preciseSECT current (eg, Forced Effect 0.6 on VIO 3; or Forced Effect 1, 10 W on VIO 300 D) to “slow cook” the vessel. Avoid cutting the vessel with DRY CUT until its lumen is clearly obliterated. For large arteries (>1 mm) and pre-emptive coagulation, use a 4 mm Coag-grasper (Olympus, Center Valley, Penn, USA) with Soft current.

For large penetrating arteries, grasp the muscle as well in the area of penetration to coagulate the intramuscular portion of the artery. This avoids having a bleeding stump that retracts within the muscle and is challenging to coagulate.

For bleeding from unclear site, use a hot biopsy forceps. It has broader contact that limits depth of thermal injury, and it can be used to grasp a larger area than the coag-grasper when there is significant bleeding that prevents precise identification of the location of the bleeding vessel. It also has a thinner shaft that allows somewhat easier aspiration of bloody fluid to improve visibility than is possible around the thicker coag-grasper catheter.

#### Myotomy tips

As demonstrated, we perform circular myotomy in the esophagus and complete full-thickness myotomy at the LES. The former increases safety in the esophagus where injury to pleura (pneumothorax) or pericardium (tamponade) remains an important safety consideration and where myotomy of the longitudinal fibers does not seem to offer significant additional benefit. At the LES, however, complete myotomy may enhance efficacy by ensuring complete disruption of the LES, and, in this area, there is less risk associated with deep penetration than in the esophagus/mediastinum.

“Pull” myotomy (distal to proximal) is preferred in patients with very tight LES in whom re-entry into the cardia after withdrawing into the esophagus to perform a traditional push myotomy (proximal to distal) may be challenging.

Progressively deeper cutting through the circular muscle with DryCut current is used for myotomy initiation. Once the proper depth is reached (visualization of the longitudinal fibers), the standard technique of “hooking” the circular fibers and cutting them can be used (at the LES, hooking also the longitudinal fibers to perform full-thickness myotomy).

Intermuscular high-pressure injection via the hybrid knife helps separate the circular muscle fibers for circular myotomy and, during full-thickness myotomy, injection of the adventitia protects against deep injury (ie, entry into the mediastinal or peritoneal cavities, which, despite not being an adverse event per se, can result in more gas escape, pain, and need for decompression).

Currents used include spray or preciseSECT coagulation in the deeper portion of the myotomy (to coagulate the “ladder” of hidden vessels just deep to the muscle), switching to DRY CUT for the more superficial relatively avascular circular muscle layer.

#### AR-POEM technique pointers

The essential feature of our AR-POEM technique is preservation of the oblique (sling) fibers of the LES. This is accomplished by a myotomy orientation and length at the EGJ and cardia that ensure preservation of these fibers. This is based on intriguing anatomic, physiological, and other data from >2 decades ago showing that the sling fibers represent the more powerful component of the LES and suggesting less GERD if care is taken to preserve these fibers during myotomy. On a posterior POEM, the oblique fibers are encountered at approximately 7 o’clock at the EGJ but then their posterior bundle follows an oblique course toward 6 o-clock , 5 o-clock, and 4 o-clock as they extend deeper in the cardia. Therefore, avoiding them during the myotomy is accomplished by a posterior myotomy orientation to the right of 6 o-clock and a myotomy length that does not exceed 2 cm into the cardia. The latter is confirmed by the double-scope transillumination technique demonstrated during the live case.

As also shown during the live case, the edge of the oblique fibers can be readily identified by the recurrent penetrating arteries recently described.^2^ These arteries are a stable anatomic feature that has also been shown in prior anatomic studies. They represent clusters of penetrating branches of the left gastric artery that start in the gastric cardia just beyond the Z line and occur at intervals of 1 to 2 cm along the border of the posterior oblique fiber muscle bundle. During the live demonstration, we exposed the first 3 such distinct clusters. We coagulated the first cluster because our 6 o-clock initial tunnel orientation at that point did not permit preservation of this cluster. However, by positioning our tunnel toward a 3 to 4 o-clock orientation as we extended it farther into the cardia, we were able to preserve the subsequent 2 clusters of penetrating vessels. As shown in this live case, this approach allowed us to completely preserve the oblique fibers located to the left of these penetrating vessels. The double scope transillumination technique confirmed a 1.8-cm gastric myotomy (distal to the Z line) at approximately a 3 o-clock orientation (opposite the gastric fundus/greater curvature at 8 to 9 o-clock orientation).

#### EndoFLIP tips

Along with other expert centers, we find the distensibility index (DI) (calculated by dividing the minimal cross-sectional area at the EGJ by the balloon pressure) to be the most useful and relevant measurement because it combines a functional measurement (pressure) and a structural one (minimal cross-sectional area). We also find useful the diameter at the tightest area of the EGJ (D_min_). D_min_ is particularly useful on the relatively rare occasions where there is a problem with obtaining a DI value, usually due to malfunction of the somewhat finicky pressure transducer of the EndoFLIP (usually caused by kinking of the catheter due to inexpert efforts to insert it through a very tight LES).

We tend to use the short EndoFLIP catheter except in selected patients with type III achalasia. This is important because we have found that the longer catheter tends to yield higher DI values than the short catheter unless the protocol is modified to measure DI at higher filling volumes than those used with the short catheter.

A standardized protocol is important. For example, in the post-POEM measurements, if one obtains higher balloon volume measurements (eg, 50 mL balloon filling) before lower ones (eg, 30 mL), the 30 mL DI measurements tend to be higher. The same occurs if longer equilibration time is allowed before taking measurements. We have performed pre-POEM and post-POEM measurements in >500 POEM patients, and therefore we are committed to the same protocol we have followed since the start. We take measurements at 30 mL and then 50 mL before the POEM, followed by 50 mL and then 30 mL post-POEM with 30-second equilibration at 50 mL. This protocol is based on our finding that, post-POEM, there may be some stretching of the fresh myotomy as the balloon is being filled, resulting in a gradual increase in DI over approximately 20 to 30 seconds.

In general, the pre-POEM distensibility in previously untreated patients is <2 (in many patients ≤1). After a conventional POEM, DI increases to approximately 6 to 8, whereas after an AR-POEM, DI increases to approximately 3 to 4. We also generally see an approximate doubling of the D_min_ after successful AR-POEM. In this live case, pre-POEM DI and D_min_ at 30 mL balloon volume were 1.7 mm^2^/mm Hg and 5.0 mm, and immediately after POEM, they increased to 4.8 mm^2^/mm Hg and 10.8 mm, respectively. We recorded measurements at 30 mL and 50 mL balloon volume as per our standard protocol applied consistently over approximately 12 years of EndoFLIP data collection from hundreds of POEMs. We generally analyze and present the 30 mL measurements^3^ as we have not found the 50 mL measurements to offer any advantage, and artifacts may occur at maximal balloon filling, including the possibility that the balloon may start to impinge on the diaphragmatic hiatus (especially in patients with a tight hiatus). This can result in measurements that may no longer reflect just the state of the intrinsic LES, which is the therapeutic target of POEM. We do provide here, however, the 50 mL measurements for reference (DI increased to 3.1 mm^2^/mm Hg from 1.3 mm^2^/mm Hg, D_min_ increased to 14.2 mm from 7.6 mm).

With time, the DI decreases from its immediate post-POEM value; this likely represents healing and remodeling that may extend beyond 3 to 6 months, which, as we have reported, may be associated with improvement in GERD. As discussed during the live transmission (Supplementary Video 1), in our published series, based on EndoFLIP data from 504 POEMs, DI increased from 1.5 to 8.5 immediately post-POEM but then decreased to 5.5 on remote post-POEM assessment at a median of 7 months.^3^ A similar trend was reported on data from 35 POEMs by the Northwestern group: pre-POEM DI of 1.7 increased to 7.0 immediately post-POEM but then decreased to 4.8 when assessed at 1 year.^4^

### Closure tips

Endoscopic suturing, even based on data reflecting our early experience with such POEM closure, had similar cost as endoscopic clips in the United States and similar time expenditure.^5^ We believe that endoscopic suturing may offer more secure closure in view of data from expert centers showing premature detachment of clips in a significant number of patients. For example, the Hamburg group, which routinely performed second-look endoscopy after POEM, reported missing clips in 9 (4%) of 238 patients 2 to 3 days after POEM, with 5 of 9 having frank dehiscence of the tunnel opening.^6^

## Post-POEM protocol

A similar protocol is used by most centers consisting of, at most, 3 to 5 total days of prophylactic antibiotics, 1 to 2 weeks of modified diet, and at least 4 to 6 weeks of proton pump inhibitor therapy (often maintained until formal GERD assessment at 3-6 months). It is not clear that routine post-POEM contrast study is required, although it is still part of our protocol.

As discussed during the live transmission (Supplementary Video 1), same-day discharge is feasible, and we use criteria for discharge similar to those reported by Zhang et al^7^: (1) American Society of Anesthesiologists (ASA) class I to III; (2) no intraprocedural adverse events; (3) secure mucosal closure; (4) postprocedure pain/nausea responsive to oral medications; and (5) patients tolerating clear fluids. Our slight caveat was using ASA class I to II instead of ASA class I to III.

## Conclusion

In conclusion, this case, which was performed during an *ASGE Live Endoscopy* course, details the technical aspects of performing an AR-POEM efficiently and safely. It includes expert commentary by the live operator and moderators of the course. Multiple practical tips and tricks are highlighted in the video and the text.

## Patient consent

The patient consented to performance of anti-reflux POEM and also consented for live transmission of the case for educational purposes.

## Disclosure

The following author disclosed financial relationships: S. Stavropoulos: consultant for Boston Scientific, Medtronic, Olympus, Lumendi, EndoQuest, and Neptune Medical. A. Bukannan: none declared.

## Supplementary data

Supplementary Video 1 Anti-reflux POEM procedure performed during ASGE Endoscopy Live: Management of Achalasia.
